# Opposition to a New Dental College

**Published:** 1892-05

**Authors:** 


ARTICLE II.

### OPPOSITION TO A NEW DENTAL COLLEGE.

The movement for the establishment of a Dental Department of the University of Buffalo is meeting with determined opposition on the part of some members of the profession, who declare that the whole scheme is "at least a generation behind the times," and would be laughed at by all advanced practitioners. The dental college plan is warmly defended by some of the ablest dentists of the city —men who are able to command large sums of money for their services as tutors of classes in our best dental colleges. The controversy is extremely interesting, and as the *Courier* presents both sides of the subject the reader may judge of its merits.

MEETING OF THE OPPOSITION.

A meeting of dental graduates of the city of Buffalo was held at the office of Dr. C. W. Stainton, No. 37 Court street. On motion of Dr. Stainton, Dr. S. Eschelman was called to the chair and Dr. W. A. Burrows chosen secretary.

The call for the meeting was read and the object stated by the president, who then called upon Dr. C. S. Butler to give a statement of the whole matter as far as he understood it. This was followed by a general discussion of the subject.

Dr. C. W. Stainton offered the following premable and resolutions, which were unanimously adopted and signed by all present.

WHEREAS Our attention has recently been directed to an announcement in the daily press of our city that the University of Buffalo has already taken steps to establish a dental department in connection with that college, and that certain measures have been adopted looking to that end which we believe to be prejudical to the best interests of that time-honored institution, as well as to dental science and the good name of the dental profession in Western New York;

THEREFORE, Believing as we do that the graduates of our reputable dental schools should interest themselves in all educational matters pertaining to our profession, as being the natural guardians of its welfare and best qualified to judge of requirements and conditions connected therewith; we, the alumni of the various dental colleges of our country, and residents of Buffalo, in convention assembled, do adopt the following resolutions, to wit:

*Resolved*, That while we fail to recognize the demand or necessary for a dental school in our city, we shall not approve or support in any manner such an undertaking unless it be founded upon correct principles, in the direction of a higher standard of education, entirely above reproach and creditable in every respect to all connected therewith.

*Resolved*, That we earnestly and respectfully request the Council of the University of Buffalo to reconsider its recent action in this matter at the earliest possible moment.

*Resolved*, That we express our unqualified disapprobation in reference to the granting of honorary degrees in dentistry, as instanced at the recent commencement exercises of the University of Buffalo, under any and all circumstances, as being in direct opposition to the spirit of the age, in conflict with the animus of our reputable dental schools, and antagonistic to the rulings and recommendations of the National Association of Dental Faculties.

*Resolved*, That a certified copy of these resolutions be transmitted to the Council of the University of Buffalo, be published also in the daily newspapers of our city, and be furnished for publication to the leading dental journals of the country.

W. V. Grove, D. D. S., Ohio College Dental Surgery, 1881.

Louis Meisburger, D. D. S., University of Pennsylvania, 1888.

S. Eschelman, D. D. S., L. D. S., Philadelphia Dental College, 1875.

J. A. Stackhouse, D. D. S., Philadelphia Dental College, 1891.

Richard Kessell, D D. S., Chicage Dental College, 1888.

H. H. Boswell, D. D. S., University of Maryland, 1884.

L. W. Robinson, D. D. S., Philadelphia Dental College, 1883.

L. S. Lodge, D. D. S., Philadelphia Dental College, 1888.

C. W. Stainton, D. D. S., Pennsylvania Dental College, 1872.

George B. Scott, D. D. S., Pennsylvania Dental College, 1875.

Cyrus A. Allen, D. D. S., University of Pennsylvania, 1881.

H. R. McMichael, D. D. S., Philadelphia Dental College, 1888.

E. J. Hausle, D. D. S., Pennsylvania College Dental Surgery, 1888.

Charles S. Butler, D. D. S., Philadelphia Dental College, 1876.

W. A. Barrows, D. D. S., Philadelphia Dental College, 1871.

REASONS FOR THE OPPOSITION.

In an interview with Dr. Stainton after the adjournment of its meeting, many points additional to those contained in the above paper were elicited. Asked what were the principal motives which produced the above protest, he answered that there were several. "First," said he, "several years since the Niagara University tried to open a dental school in connection with their medical college here. The scheme was fully as advanced as this new attempt and even more so—but the opposition of the dentists was such that it was given up. At the principal meeting against it a resolution was adopted that hereafter no effort should be made to start a dental school unless it had the unqualified support of the dental profession here. The man who offered that resolution has been the principal factor in this new attempt and has violated entirely that agreement.

"There has been no respect paid to the wishes of the dentists here. They have not been consulted. Sometime in April, 1891, about a dozen of us dentists got together and the result of this meeting was the appointment of a committee of five to consider the matter and report at some future time.

"They have never reported. Three of the committee got disgusted and dropped out. The others have gone and organized a school without regard to the wishes of any one of their dental brethren except themselves. They have taken the chief positions and of course are complacent.

"Second, there is no general conviction that such a school is needed here or would be of benefit to the profession or to the University. There has been a craze to organize dental schools for some years past. Chicago has about 30 charactered schools; many of them, of course, are yet only on paper. But it shows how the fever has spread. If not another dental school were started for 25 years it would be one of the best things that could happen for the cause of dental education. I know that not one of the men interested in the starting of this new dental school, nor one of the men whose names are attached to the above protest, have a dental student or have had one for years. To talk of the need of a school under this condition seems insincere.

"Third, the chief disapprobation felt, is because the school has been started by methods no longer tolerated in dental circles, and which will almost inevitably prevent its being a school of reputable character."

When asked to explain this latter assertion more fully Dr. Stainton said: "In August, 1884, the faculties of the various dental colleges in the United States associated together under the name of the "National Association of Dental Faculties." They lay down all rules as to admission of students to colleges, their length of pupilage, and the conditions of their graduates. Every dental college and every dental department of any University must receive recognition by this body before its diplomas are received by the examining board of any State of the Union. Year by year this body of educators has been raising the status of dental graduation and examining more carefully the character of new dental schools.

"Among some of the abuses they laid hands on years since was the granting of honorary degrees. 'The National Board of State Examiners, an auxiliary body, had refused In 1883 to honor the diploma of any school guilty of granting honorary degrees. In 1889 the Baltimore Dental College, the oldest dental school in the world, asked leave of the National Association to grant the degree of D. D. S. honorably on a prominent practitioner, but the request was denied. In several instances colleges have been suspended for attempting to graduate students in less than the time prescribed.

"Yet here in Buffalo is an attempt to start a school by methods at least a generation behind the age. Only one of the announced faculty has received his dental diploma in the manner approved for years by our highest authority. The chief agitator received his D. D. S. without attending any dental college, and his two chief supporters have had the degree conferred upon them by the 'University of Buffalo,' which never conferred the degree before, and without any pretense of attending lecture. The thing is so ridiculous that it will be a laughing stock in every center of dental intelligence in the country. All dental colleges and all dental departments of universities must have one full scholastic year before they can make application to the executive committee of the National Association, and this application must be made 60 days before the annual meeting of the Association, which is in August of each year. If this executive committee report favorably on the application it lies over one year before it is acted on. So that this Buffalo school cannot receive any official standing before August, 1894. We feel very confident that when the executive committee knows the facts in the case as it will know them, no such recognition can be had."

When inquiry was made as to the feeling for or against the school among the reputable practitioners of dentistry here, the Doctor answered by saying that no attempt had been made to canvass this matter except among graduates in actual practice, who have been graduates long enough to get them into the city association. The protest is signed by every such person except one—who is in the new faculty.

"The feeling is so determined on the part of the signers of the protest that marked copies of the *Courier* will be in the hands of every prominent dentist in this country, and in the hands of the dean of every dental college in the country in a few days."

### THE COLLEGIANS' SIDE.

Dr. W. C. Barrett, the distinguished dental surgeon and a lecturer of the University, was visited at his home by a representative of the *Courier*, that the story of the proposed dental department might be heard from the standpoint of one who has interested himself in it. Dr. Barrett, who had lately returned from Chicago, where he was required to perform some arduous labors in lecturing before a class of students, was ill in bed, but kindly consented to grant an interview to his visitor "if his mission was important." Dr. Barrett said:

"For a good many years the University has been desirous of making its medical teaching complete. To do this requires four departments, medicine, pharmacy, dentistry, and veterinary medicine. The University has had two of these departments for some time, namely, medicine and pharmacy, but long before the establishment of the latter department the Council had taken steps to establish a dental school. Owing to various causes, such as lack of funds, want of harmony; and the absence of proper accommodations in the University building, nothing had ever been accomplished. With the erection of the new University building, the medical faculty thought the time had come for the establishment of a dental department. The plans for the building were drawn, and included the necessary room, before the dentists were specially approached. Then the matter was laid before some of them and they were informed that if the dentists of Buffalo did not choose to take the responsibility, others would be called upon, for the Council was determined to complete the original plans. Consultations were held which finally resulted in calling a meeting of the principal dentists of the city. That meeting voted that it was expedient to open a dental department, and a committee of five was appointed with power to carry out the objects of the meeting. The committee met and held consultations, but the members could not agree upon a definite course of action, and finally referred the matter to a sub-committee of two. That sub-committee formulated a plan and submitted it to the members of the committee of five, who in general terms approved it.

"The first acknowledged step that was taken resulted in the development of an unexpected opposition. The sub-committee consulted with the medical faculty and has endeavored to be guided by their counsel. They realize the necessity for entire harmony of action among those who are to take an active part in the school, and therefore have called into their councils only such as they felt had the same ends and aims in view as themselves. They realize that dissension in their ranks would mean ruin in their undertaking. There was nothing to be made out of it, on the part of any one, for the work that was done must for a long time, at least, be without the hope of any compensation; and the money that they would necessarily expend must be for the benefit of the University, of the dental profession, and the city of Buffalo. The college, when organized, would belong to the University, and its belongings must be vested in it, so there was no chance for any pecuniary return.

"But those engaged in the undertaking had a pride in the city of their residency, a love for their profession, and a desire to do something to build up institutions of learning, Therefore they were willing to expend their money and give their time and undertake the labor necessary for what they thought was for the best interests of all concerned.

"At the late commencement of the University four gentlemen were appointed by the Council as the governing board for the new department. They will associate with themselves such help as is needed, and if it cannot be found in Buffalo, it can easily be procured elsewhere. The department will be opened this fall, for they have pledged themselves to the University that this shall be done, and have received assurances of the hearty support, not only of the Council of the University, but of the other departments.

"At the late commencement the honorary degree of Doctor of Dental Surgery was conferred upon two dentists concerning whose standing there can be no question. One of these is the oldest practitioner in the city. He has been the State Censor for this district for 15 years or more and has been prominently identified with professional movements in the State. The other is a practitioner of acknowledged eminence and skill, a prominent dental society man, and a leader in all professional movements. Neither possessed the degree of the schools, for they had entered upon practice before the modern era of dental education had its inception. The University honored itself in conferring these degrees, for the degrees of the most of the oldest practitioners are honorary degrees. Twenty-five years ago there were not more than four or five dental colleges in existence in the world, but with the passage of the laws regulating dental practice a dental diplôma has become a necessity for a practitioner.

"Of course it is a matter of regret that any opposition should have been aroused. None but perfectly legitimate means have been employed. The school will come up to the highest requirements of dental practice and will be made as perfect in its teaching and equipments as the abilities of those engaged will permit. It would seem that nothing could be done in Buffalo for the furtherance of general or professional interests without arousing opposition. Nothing has been attempted for the beautifying of of the city or for the advancement of any public interest without arousing the ire of the obstructionists. Prof. Park, in a letter to one of these, expressed the truth in a nutshell when he said: 'If your objections are not well founded, as it seems to me is the case, it shows that you are too much like a great many of Buffalo's better citizens who have little or no pride in Buffalo institutions, and who are unwilling to do anything by which they may be built up or improved. I should be sorry to think that there is any one who does not want the University of Buffalo to advance and be in fact what it is in name.'

"It seems a pity that there should be any one who, if unwilling themselves to do anything to forward Buffalo's interests, should possess a desire to hinder others in so good a work. The School of Dentistry is an assured fact. The gentlemen connected with it propose to make of it all that it is possible for them to do. If their professional brethren choose to hinder the work, to embarrass their efforts, and to prevent the making of the school all that it otherwise might be, they can only deeply regret it. Their course is plain before them. They have pledged themselves to a distinct line of effort and they will carry it out to the very best of their ability.

